# Editorial: Evolution and diversity of avian gut microbiomes

**DOI:** 10.3389/fmicb.2023.1348762

**Published:** 2023-12-20

**Authors:** Kasun H. Bodawatta, Michael Kogut, Michael W. Taylor

**Affiliations:** ^1^Section for Molecular Ecology and Evolution, Globe Institute, University of Copenhagen, Copenhagen, Denmark; ^2^USDA-ARS, Southern Plains Agricultural Research Center, College Station, TX, United States; ^3^School of Biological Sciences, University of Auckland, Auckland, New Zealand

**Keywords:** bird gut microbes, host-microbe associations, evolution, host physiology, symbionts

Birds represent a diverse class of vertebrates that exhibit a fascinating array of adaptations and behaviors, while playing vital ecological roles across the globe as pollinators, seed dispersers, and top predators. Their gut microbiomes may facilitate these ecological roles through influencing the physiology, metabolism, and immune functions (Bodawatta et al., 2022). A recent surge in studies on avian gut microbiomes has broadened our understanding of the drivers of these complex microbial communities and provided insights into how gut microbes facilitate bird adaptations. This timely Frontiers Research Topic, thus, provides a diverse collection of recent advances in the field of avian gut microbiomes, with insights into processing microbial samples, avian gut viromes, microbial shedding and the influence of gut microbes on host health and physiology.

This Research Topic contains seven articles involving 53 authors from a diversity of research institutes. Six of the articles focus on bacterial communities, while one article focuses on gut viral communities, providing novel insights into an understudied aspect of the avian microbiome. Studied host species span across the avian phylogeny including ratites (e.g., ostrich, kiwi), galloanserines (e.g., chicken, duck, goose), raptors (vultures) and passerines (Japanese tits). The majority of the work was conducted in captive bird species, while four studies have conducted manipulation work to delve deeper into the functions of gut microbes. This diversity of focal taxa and research approaches highlights the positive trajectory of avian gut microbial research to thoroughly understand these complex host-microbe systems.

Four articles in this Research Topic use commercial bird species such as chickens, ducks and geese to investigate how gut microbiomes influence host health and physiology. First, Sun et al. illuminate how bacteria producing short-chain fatty acids may influence residual feed intake and feed efficiency in laying ducks, proposing a potential role for specific bacteria in regulating energy for improved feed utilization efficiency. Second, Campos et al., show varied responses of microbiota in the duodenum and jejunum toward *Eimeria acervulina* infections, with potential implications for short-chain fatty acid production. Third, Song et al. highlight the potential therapeutic role of gut microbiota, particularly *Bacteroides xylanisolvens* as an effective anti-hyperuricemia bacterium, in addressing hyperuricemia and gout in goslings. Finally, Li et al. demonstrate that bile acid supplementation proved effective in enhancing growth performance, lipid metabolism, intestinal health, and cecal microbiota structure in geese. Overall, these studies provide novel insights into defensive and physiological ramifications of intricate host-gut microbe associations in birds.

The remaining three articles cover a range of Research Topics aimed at gaining insights into avian gut microbiomes. Edwards et al. pinpoint that despite variations in efficacy of storage and extraction methods, the resulting 16S rRNA gene-based bacterial community profiles remain robust within bird species across the phylogeny, providing valuable insights for future research on the avian gut microbiota. Xin et al. provide evidence of host modification of microbiomes in their nest environments, by shedding more potentially beneficial bacteria. The final article of this Research Topic focuses on gut DNA viromes of Himalayan vultures. Here, Zhai et al. identify complex and diverse viral communities in vulture guts, emphasizing variations between wild and captive states and providing a baseline for future research on vulture rehabilitation.

The composition of avian gut microbiota is influenced by a number of factors, including host genetics, lifestyle and diet (Bodawatta et al., 2022). This Research Topic attempted to shed further light on the evolution and diversity of avian microbiomes, and how environmental factors can shape avian gut microbiome functions and structures, as well as the influence of the microbiome on avian physiology. The breadth of topics and study host species across the assembled articles in this Research Topic speak to the dynamic nature of this burgeoning field. Avian microbiome research, while undeniably still the little sibling to the much larger and better-established theme of mammalian (especially human) microbiomes, is nevertheless on an exciting upward trajectory.

## Author contributions

KB: Writing – original draft, Writing – review & editing. MK: Writing – original draft, Writing – review & editing. MT: Writing – original draft, Writing – review & editing.

## References

[B1] Bodawatta K. H. Hird S. M. Grond K Poulsen M. Jønsson K. A. (2022). Avian gut microbiomes taking flight. Trends Microbiol. 30, P268–P280. 10.1016/j.tim.2021.07.003 34393028

